# Modifications in the structure of the lichen *Cladonia* thallus in the aftermath of habitat contamination and implications for its heavy-metal accumulation capacity

**DOI:** 10.1007/s11356-017-0639-1

**Published:** 2017-11-05

**Authors:** Piotr Osyczka, Piotr Boroń, Anna Lenart-Boroń, Kaja Rola

**Affiliations:** 1 Institute of Botany, Faculty of Biology and Earth Sciences, Jagiellonian University, Kopernika 27, 31-501 Kraków, Poland; 2 Department of Forest Pathology, Mycology and Tree Physiology, University of Agriculture in Kraków, 29 Listopada Ave. 46, 31-425 Kraków, Poland; 3 Department of Microbiology, University of Agriculture in Kraków, Mickiewicza Ave. 24/28, 30-059 Kraków, Poland

**Keywords:** Lichenized fungi, Phenotypic plasticity, Heavy metals, Bioaccumulation, Oxalate crystals, Biomonitoring, Thallus anatomy

## Abstract

Phenotypic traits of lichens can be greatly modified by environmental factors. Granulose thalli on soil and podetia, densely covered with granules, referring to common and widespread lichen *Cladonia cervicornis* subsp. *verticillata* were found near zinc smelter. The granules are stratified, filled with fungal medulla and heavily encrusted with calcium oxalate weddellite crystals, not observed on regularly developed thalli of the species. Phylogenetic analysis revealed that deformed granulose forms belong to this taxon, showing that the phenotypic plasticity of the lichens of *Cladonia* can lead to the emergence of features that do not coincide with the taxonomic definition of the species. The heavy-metal accumulation capacity of both granulose and regular form of primary and secondary lichen thallus, in relation to the element content in corresponding substrate, was determined. Granulose-modified thalli accumulate greater amounts of heavy metals than regular ones, meaning that the bioaccumulation property of a given species may be greatly affected by morphological modifications. The granulose forms are also characterised by considerably higher ratios of Cd, Pb and As concentrations in lichen samples in relation to the corresponding substrates than regular ones. This means that collection of variously formed thalli should be avoided in biomonitoring sampling procedures. The results indicate that a substantial part of the element load, in particular zinc, in the examined lichen thalli collected near the smelter originates from atmospheric fallout.

**Electronic supplementary material:**

The online version of this article (10.1007/s11356-017-0639-1) contains supplementary material, which is available to authorized users.

## Introduction

Lichens are a symbiotic association composed of a lichen-forming fungus and an alga and/or cyanobacterium (Ahmadjian 1993). Lichens lack a root system and other absorptive organs as well as protective cuticles and filtration mechanisms. Therefore, both necessary nutrients and toxic elements, dissolved in the atmosphere as well as present in the substrate, can be absorbed through the whole surface of the thalli (Tyler 1989; Bačkor and Loppi 2009). The relative sensitivity of lichens to various contaminants, such as sulphur dioxide, heavy metals, radionuclides and organic impurities, depends on fungal biology and the nature of photosynthesis contributors (Beck 1999; Nimis et al. 2002; Guschina and Harwood 2006). Contamination usually leads to a decrease in biodiversity and impoverishment of lichen biota (van Haluwyn and van Herk 2002; Sujetovienė 2015), but several tolerant or insensitive species, benefitting from a low level of competition, frequently increase in abundance (Nash and Gries, 1991; Wolseley et al. 2006; Rola and Osyczka, 2014; Rola et al. 2014, 2015). Local environmental conditions, such as insolation, humidity and thermal fluctuations, as well as various kinds of anthropogenic stressors, often modify the organisation and structure of lichen thalli (Pintado et al. 1997). Both field observations and laboratory experiments have documented various lichen responses to pollutants (e.g. Palomäki et al. 1992; Carreras et al. 1998; Tarhanen et al. 1999; Zambrano and Nash 2000; Carreras and Pignata 2002; Bajpai et al. 2013; Nakajima et al. 2015; Paoli et al. 2016). Noticeable modifications of external morphology, ultrastructural changes, disturbances in physiological processes, accumulation of calcium oxalate and a decrease in the potential for sexual reproduction at the intraspecific level may occur (see Nash and Gries, 1995; Otnyukova 1997; Cuny et al. 2004; Paoli et al. 2015; Mateos and González 2016). Impaired thallus development, convolution, bleaching or changes in coloration, detachment from the substratum, chlorotic or necrotic patches and changes in layers thickness in cross section are among the first visible signs of a lichen’s reaction to pollution (Goyal and Seaward 1982; Sigal and Nash 1983; Scott and Hutchinson 1989; Otnyukova 2007). Growth abnormalities were found, inter alia, in individuals of *Cladina* (subgenus within *Cladonia*) even at considerable distances from the main source of SO_2_ pollution, and all morphological abnormalities were correlated with thallus sulphur concentrations (Otnyukova 1997).

Lichens of *Cladonia* are widespread throughout the world and characterised in principle by morphological dimorphism; they first produce a primary thallus, usually in the form of squamules, and then variously formed erect secondary thalli, called podetia (Ahti 2000; Ahti et al. 2013). Habitat factors often modify the structure of their thalli (Kotelko and Piercey-Normore 2010; Pino-Bodas et al. 2010; Osyczka and Rola 2013b) and the ability of some species to adapt to various microhabitats is frequently manifested by clear eco-morphological differentiation (Burgaz et al. 1993; Osyczka et al. 2007; Osyczka et al. 2014). Numerous species appear to be effective and rapid colonisers of bare ground in both natural and anthropogenic habitats, such as poor grasslands, wastelands and post-industrial areas (Paus 1997; Cuny et al. 2004). Some *Cladonia* species are very well adapted to high contamination and play a fundamental role in the natural restoration of strongly affected habitats. Relatively low bioaccumulation, in combination with a restrained heavy-metal accumulation pattern as well as a decrease in metal content along the vertical gradient of thalli, has been considered an important attribute of *Cladonia* in the colonisation of highly contaminated sites (Osyczka and Rola 2013a; Osyczka et al. 2016). There are also several species less resistant to heavy substrate contamination; nevertheless, they are first to inhabit disturbed and human-transformed poor psammophilous grasslands (Rola and Osyczka 2014).

One of the widespread, locally common, and morphologically distinctive boreo-montane species is *C. cervicornis* subsp. *verticillata* (Hoffm.) Ahti [*C. verticillata* (Hoffm.) Schaer]; for detailed description see van Herk and Aptroot (2003) and James (2009). This is a typical epigeic lichen confined to dry and open places such as grasslands, heaths, sand dunes and mine spoil heaps. During a study on specific assemblages of cryptogams inhabiting psammophilous grasslands around a zinc factory (see Rola and Osyczka 2014), unknown lichen thalli were found in the form of densely packed corticated granules growing directly on soil. Simultaneously, similar granules were observed on podetia clearly referring to *C. cervicornis* subsp. *verticillata*. Interestingly, this zone constitutes the local distribution border beyond which this *Cladonia* lichen is no longer able to grow.

The primal purpose of the study was to determine the status of modified lichen thalli; four different scenarios explaining the nature of these granules were taken into consideration: (1) the granules represent strong morphological modifications of *C. cervicornis* subsp. *verticillata* thalli; (2) the granules constitute a separate entity which exists independently and is parasitic on *C. cervicornis* subsp. *verticillata*; (3) the granulose specimens form a separate lineage, but one closely related to *C. cervicornis* subsp. *verticillata*; (4) alternatively, the granulose structure of thalli is associated with the presence of an atypical photosynthetic partner. Additionally, lichen material and the corresponding substrate were examined in terms of heavy-metal content. We wanted to verify whether atypical granulose form of thallus differs in heavy metal accumulation capacity from regular form and thereby to recognise the effect of changes in the thallus structure on its accumulation capacity. We set two fundamental hypotheses: (1) pollution resulting from industrial activity modifies the growth form of lichen thalli to a remarkable extent, and the peculiar corticated granules in fact represent *C. cervicornis* subsp. *verticillata*; if yes, (2) the granules on soil and podetia covered by granules exhibit a higher heavy-metal accumulation capacity than typical primary squamules and regular podetia of *C. cervicornis* subsp. *verticillata*.

## Materials and methods

### Study area

The lichen samples were gathered from psammophilous grasslands within the Pustynia Starczynowska desert (the Silesia-Cracow region, southern Poland) inhabited by a large population of *C. cervicornis* subsp. *verticillata*. The exposure of a vast sandy area in combination with a reduction of groundwater level has led to the development of specific communities (Rahmonov and Oleś 2010) with a clear predominance of cryptogamic organisms and a negligible contribution of vascular plants (Rola and Osyczka 2014). At present, the desert surrounds the zinc smelter ‘ZGH Bolesław’ Mining and Smelting Works. It has operated for over 60 years, and emissions of pollutants, including heavy metals and SO_2_, have resulted in a high level of soil contamination in the adjoining area (Liszka and Świć 2004; Danek 2007). Dustfall from this smelter was among the highest such falls in the country, reaching 500 t per year (Szarek-Łukaszewska 2009).

### Material sampling

Ten sampling sites were randomly selected 2200 to 3400 m from the smelter. According to Rola and Osyczka (2014), this distance range corresponds to the zones 2 and 3 that are characterized by similar heavy metal concentrations in soil. Four different morphological types of lichen samples were collected within each site; they represent two kinds of thallus, i.e. primary and secondary, and two forms of thallus, i.e. granulose and regular. Simultaneously, top layer of soil substrate to a depth of 5 cm were gathered from each site. For comparison purposes, lichen and soil materials were collected from reference area (the Pustynia Błędowska desert), which also constitute psammophilous grassland habitat but is located at the distance of 10 km from the smelter. Details on lichen material are provided in Table 1.

**Table 1:** Lichen material and number of examined samples

Abbreviation	Lichen samples	Kind of thallus	Form of thallus	Number of samples	
				Pustynia Starczynowska desert	Pustynia Błędowska desert (reference area)
Re-Pr	Regular squamules of *C. cervicornis* subsp. *verticillata*	Primary	Regular	10	3
Gr-Pr	Thoroughly epigeic corticated granules	Primary	Granulose	10	Not found
Re-Pd	Typically formed smooth regular podetia of *C. cervicornis* subsp. *verticillata*	Secondary	Regular	10	3
Gr-Pd	Podetia referring to *C. cervicornis* subsp. *verticillata* densely covered with granules	Secondary	Granulose	10	Not found

### Lichen samples determination

The Gr-Pr, Gr-Pd and Re-Pd lichen samples were identified molecularly with ITS barcode sequence; both mycobiont (primers ITS1F and ITS5—White et al. 1990) and photobiont (primers ITS1T and ITS4T—Kroken and Taylor 2000) were analysed. All details concerning sample preservation and storage, DNA extraction and PCRs were carried out according to procedures presented in Osyczka et al. (2014). Bidirectional sequencing of PCR products was carried out with the use of PCR primers. Sequencing was performed with the BigDye Terminator v3.1 Cycle Sequencing Kit (Life Technologies, USA) on a T100 thermal cycler (Bio-Rad) and 3500 Series Genetic Analyser (Life Technologies, USA) using standard protocols. The acquired sequences were queried against NCBI GenBank with BLAST search tool to determine the most closely related accessions. Each lichen sample was analysed for secondary metabolites determination using thin-layer chromatography (TLC; solvent systems C and G), according to the standardized method summarized by Orange et al. (2001).

### Micro-morphological and anatomical observations

The unwashed and air-dried lichen samples representing both kinds and forms of the thallus were observed under stereoscopic microscope and by scanning electron microscopy (SEM) using a HITACHI S-4700 and NORAN Vantage after sputter-coating with a thin layer of gold for micro-morphological observations or carbon for elemental analysis by the energy-dispersive X-ray spectroscopy (EDX). The size of Gr-Pr was assessed on the basis of measurement of 100 random granules. The Gr-Pr and Gr-Pd samples were sectioned with a rotary microtome (Microm, Adamas Instrumenten) and stained with a lactophenol blue solution for observation under a light microscope.

### Chemical analysis

Substrate acidity (pH) was electrometrically determined in 1 M KCl suspensions with a Hach Lange HQ40d pH metre. Organic carbon content was measured via dry combustion technique with a LECO SC-144DR analyser and total N content by the Kjeldahl method with a Kjeltec 2300 Analyzer Unit (Foss Tecator).

Total concentrations of Cd, Pb, Zn and As (referred to herein as heavy metals) were determined in the lichen and substrate samples. These elements are the main contaminants associated with the processing of zinc and lead ores and total concentration of elements in relation to lichen accumulation are routinely included in the procedures for biomonitoring studies (see Garty 2002). Additionally, total Ca content was measured in soil samples to verify whether alternatively the calcium enrichment of the habitat is not a potential stimulus for calcium oxalate production by lichen individuals. The substrate samples were dried and passed through a 2-mm sieve. Then 2 g DW were digested in 70% HClO_4_ (Merck, Suprapur). Macroscopic foreign materials adhering to thalli surfaces were carefully removed with plastic tweezers. Moreover, lichen samples were rinsed with deionised water to remove finer particulate matter (cf. Naeth and Wilkinson 2008; Bačkor et al. 2010; Pawlik-Skowrońska and Bačkor 2011) and then dried at 90 °C for approximately 24 h to a constant weight. Dry and powdered lichen samples with an average weight of 50 mg for Re-Pr and Gr-Pr and 100 mg for Re-Pd and Gr-Pd were digested in 70% HClO_4_ (Merck, Suprapur) and 65% HNO_3_ (Merck, Suprapur) (1:4) and diluted with double-distilled water. Concentrations of particular elements were determined by means of atomic absorption spectrometry using a Varian AA280FS and Varian AA280Z with a GTA 120. Certified standard solutions (Merck, Titrisol) were used to prepare the elemental calibration standards and quality assurance. Analyses of elements were repeated at least three times. Additionally, analytical precision was checked against certified reference materials (CRM048-50G, INCT-OBTL-5) and samples fell within ± 10% of the certified value.

### Statistical and phylogenetic analyses

The bioaccumulation of the elements for particular types of lichen samples in relation to their content in the substrate were calculated according to the formula: concentration of the element in the lichen thallus ÷ concentration of the element in the corresponding substrate (specified here as CL/CS factor). In a sense, this is equivalent to the standard bioaccumulation factor (BAF; concentration in an organism ÷ concentration in the ambient environment). Nevertheless, the main source of the elements in the lichen thalli (substrate or atmospheric fallout) is not unambiguous; therefore, we avoided using the term BAF directly during our study.

Two-way analysis of variance (kind of thallus × form of thallus), followed by Tukey’s (HSD) test, was performed to reveal significant differences in the element concentrations and CL/CS factor values across particular types of lichen samples. Prior to the analysis, the distribution normality was verified using the Lilliefors test. Levene’s test was performed to assess the equality of variances.

Factor analysis, using PCA for factor extraction, was used to reveal relationships between examined soil parameters. Factors with eigenvalues > 1 were chosen according to the Kaiser criterion and then varimax rotation was applied. The analyses were carried out using STATISTICA 12 (Statsoft, Tulsa, OK, USA).

Bayesian inference was used to analyse the variation among ITS sequences. First all acquired ITS sequences were aligned along with 5 *C. cervicornis* subsp. *verticillata* GenBank accessions. Additional *C. rei* and *C. subulata* sequences were used as a two level outgroup. Alignment was prepared with the ClustalW algorithm (Thompson et al. 1994) of MEGA 6 suite (Tamura et al. 2013), inspected visually and corrected manually if necessary. In this form alignment was tested for best fitted evolutionary model with jModeltest (Darriba et al. 2012) limiting the number of substitution schemes to three to accommodate only models implemented in MrBayes. In this process the best fitted, according to BIC criterion, model for Bayesian analyses was designated. Next, the gap matrix was generated and MrBayes input file was prepared with FastGap 1.2 (Borchsenius 2009). Both datasets, aligned sequences and binary gap matrix, were analysed jointly using Bayesian inference with MrBayes 3.1.2. (Huelsenbeck and Ronquist 2001). Analysis was run until the standard deviation of split frequencies stopvalue of 0.01 was reached. The 50% majority tree generated during the analysis was visualised in MEGA 5.1.

## Results

### Variation of ITS sequences

Mycobiont ITS sequences were generated for 26 samples; GenBank accession nos. KY054855-KY054880. According to the NCBI GenBank, all sequences representing the Re-Pr, Gr-Pr, Re-Pd and Gr-Pd samples confirmed their affinity to *C. cervicornis* subsp. *verticillata*. Photobiont ITS products were generated and sequenced for eight *C. cervicornis* subsp. *verticillata* samples and one *C. phyllophora* sample (see Fig. S1). All detected photobionts proved to be genetically uniform on the ITS level and to belong to the *Asterochloris* genus based on comparison with GenBank sequences (for example: AF345392, AY622840, FM945351) acquired from different *Cladonia* species.

The two outermost clades of phylogenetic tree are occupied by outgroup species, the outermost comprised of *C. rei* and *C. phyllophora* sequences, the next-outermost comprised of *C. subulata* sequences (Fig. S1). This two-layer outgroup base contains the root for the strongly supported (posterior probability: pp = 95) inner clade that groups all our *C. cervicornis* subsp. *verticillata* sequences, regardless of their type (Re-Pr vs Gr-Pr vs Re-Pd), with all *C. cervicornis* GenBank accessions representing different parts of the world. Additionally, samples of all kinds and forms of thallus, including those with detected *Asterochloris* photobionts, are scattered among all subclades of the *C. cervicornis* clade.

### Morphological and anatomical structure

**Fig. 1:**
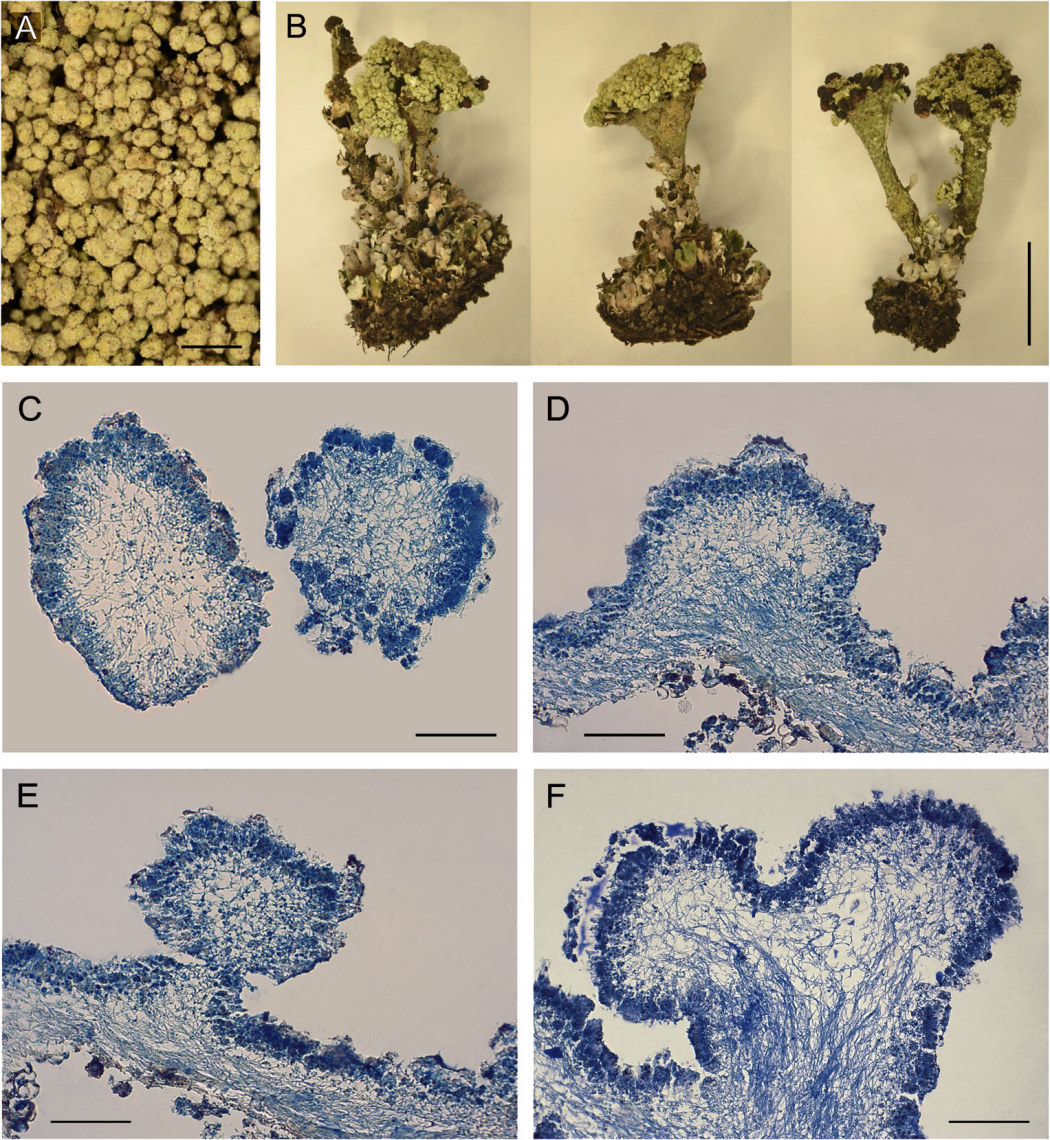
Modified forms of *Cladonia cervicornis* subsp. *verticillata* with granulose primary thallus and podetia (secondary thallus) covered with granules. **a** Corticate granules on soil, scale = 1 cm. **b** Podetia covered with corticate granules in upper parts and inside the scyphi, scale = 1 cm. **c** Cross section of corticate granules on soil, scale = 150 μm. **d**–**f** Cross section of parts of podetia covered with corticate granules, scale = 150 μm

**Fig. 2:**
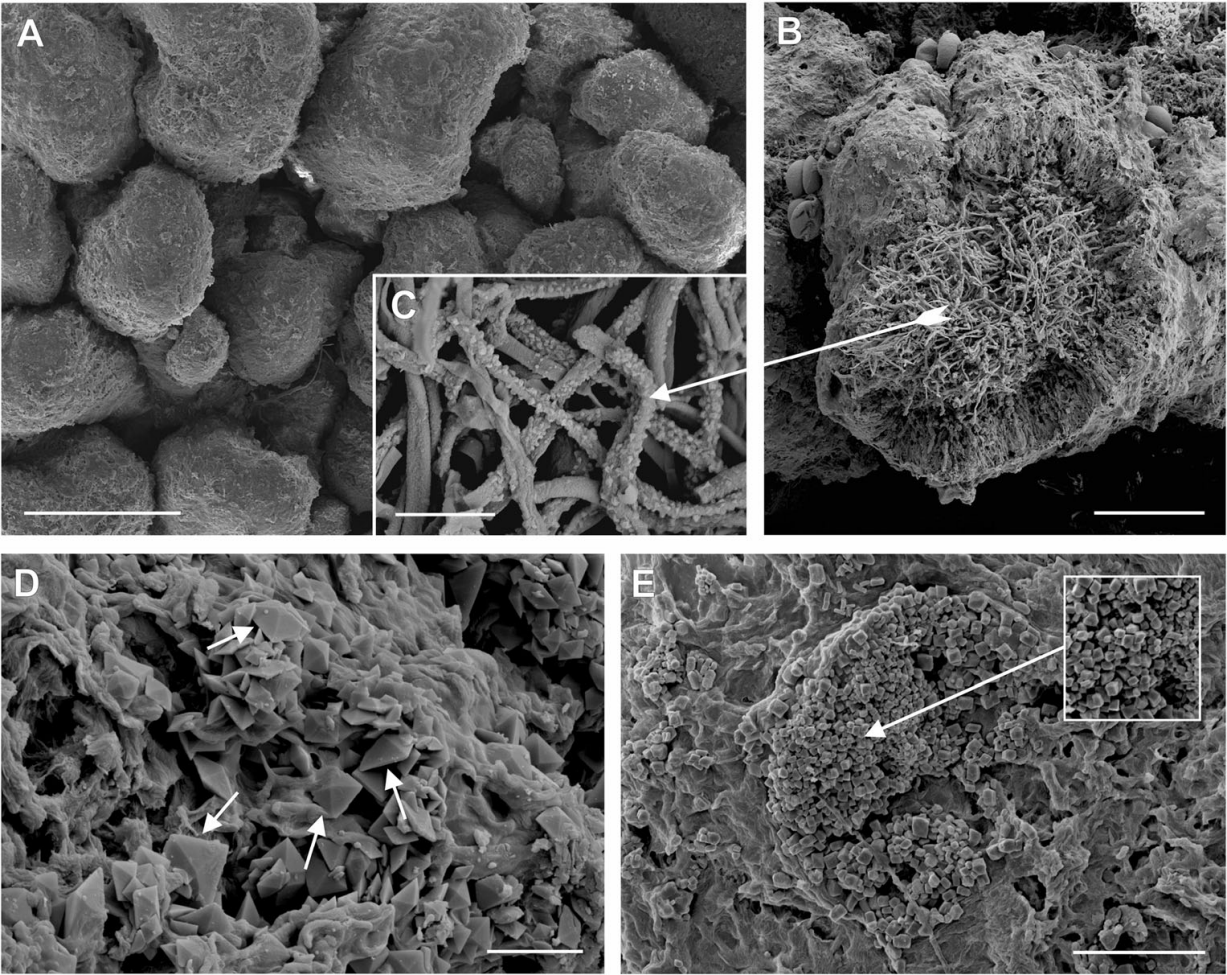
SEM micrographs of corticated granules presenting modified forms of *Cladonia cervicornis* subsp. *verticillata*. **a** General view of corticate granules on soil, scale = 0.5 mm. **b** Interior of granule; fungal hyphae of the medulla and outer cortex are visible, scale = 100 μm. **c** Fungal hyphae inside granules within the medulla encrusted with small crystals, scale = 10 μm. **d** Calcium oxalate di-hydrated weddellite in the form of bipyramids deposited on the surface of granules, scale = 10 μm. **e** Calcium oxalate di-hydrated weddellite in the form of short tetragonal prisms concentrated and forming crater-like structures on the surface of granules, scale = 25 μm

Densely packed granules growing directly on soil are single or divided irregularly and covered by a discontinuous cortex. Their size ranges from 0.18 to 0.88 mm (Fig. 1a, c); the average size is 0.49 mm. Analogous structures observed on podetia, especially at the upper part (Fig. 1b), are formed by the overgrowth of the medulla layer (Fig. 1d, f). The anatomical structure of granules is clearly stratified and completely filled with the fungal medulla (Figs. 1c–f, 2b). The granules can be detached from the parental podetium (Fig. 1e). Irrespective of kind and form of thallus, all lichen samples were chemically identical and contained fumarprotocetraric acid as a major and protocetraric acid as an accessory substance.

Microscopic investigation of the granules using SEM (Fig. 2a) disclosed the presence of heavy crystalline deposits on their upper surface and directly on the internal fungal hyphae (Fig. 2c). The crystals varied in size, but those on the surface were of a regular shape corresponding to bipyramids (Fig. 2d) and short tetragonal prisms (Fig. 2e). The crystal morphologies and the element counts obtained by means of the EDX system integrated with the SEM justify the definition of both crystal forms as representatives of calcium oxalate di-hydrated weddellite (COD; CaC_2_O_4_ · (2 + *x*) H_2_O). The crystals were singly dispersed on the surface or grouped into irregular aggregates and crater-like structures (Fig. 2e). In addition to calcium abundance, the EDX detection revealed lead enrichment and a trace of sulphur on the surface of the crystals and in the area of their aggregations (Fig. S2). Crystalline production was characteristic only for the granules and the deposition was particularly abundant on/within the Gr-Pr samples. The samples of typically developed primary squamules and podetia did not demonstrate a tendency towards calcium oxalate deposition.

### Heavy-metal content in soil and lichen samples

The soil chemical parameters could be regarded as unfavourable for vegetation. The pH was acidic to slightly acidic, the Ca content very low and the soil fertility poor in terms of the content of organic carbon and total nitrogen. The relationships between particular soil parameters were revealed using factor analysis (Table 2). Three factors explained 84.71% of the total variation. The first factor represents pH and contaminant elements such as Zn and Cd; the second factor included total C, Ca and Pb; the last factor is, remarkably, associated only with As.

**Table 2:** Soil chemical properties of the Pustynia Starczynowska desert and factor loadings (varimax-rotated) of these properties determined by factor analysis for the examined sites. Loadings greater than 0.7 are shown in bold. Soil characteristics for the reference area (Pustynia Błędowska desert) are also provided

Parameter	Pustynia Starczynowska desert						Reference area
	Min	Max	Mean ± SD	Factor 1	Factor 2	Factor 3	
Explained variance (%)				39.61	32.58	12.52	
pH_KCl_	4.11	5.36	4.50 ± 0.41	**0.913**	0.172	− 0.099	5.57 ± 0.39
Total C (%)	0.25	1.38	0.85 ± 0.37	− 0.207	**0.927**	0.035	2.66 ± 0.38
Total N (%)	0.02	0.07	0.04 ± 0.02	0.626	0.471	0.314	0.17 ± 0.06
Total Ca (mg kg^−1^)	114.53	210.82	161.97 ± 38.37	0.487	**− 0.781**	− 0.191	1216.29 ± 63.19
Total Cd (mg kg^−1^)	1.83	3.40	2.59 ± 0.52	**− 0.931**	0.229	− 0.152	1.56 ± 0.14
Total Pb (mg kg^−1^)	50.42	192.50	111.04 ± 45.14	0.342	**0.848**	0.031	251.49 ± 17.72
Total Zn (mg kg^−1^)	69.83	225.69	144.98 ± 54.77	**− 0.785**	0.201	0.037	167.56 ± 59.76
Total As (mg kg^−1^)	2.76	7.31	5.25 ± 1.75	0.021	0.097	**0.978**	4.48 ± 0.63

**Fig. 3:**
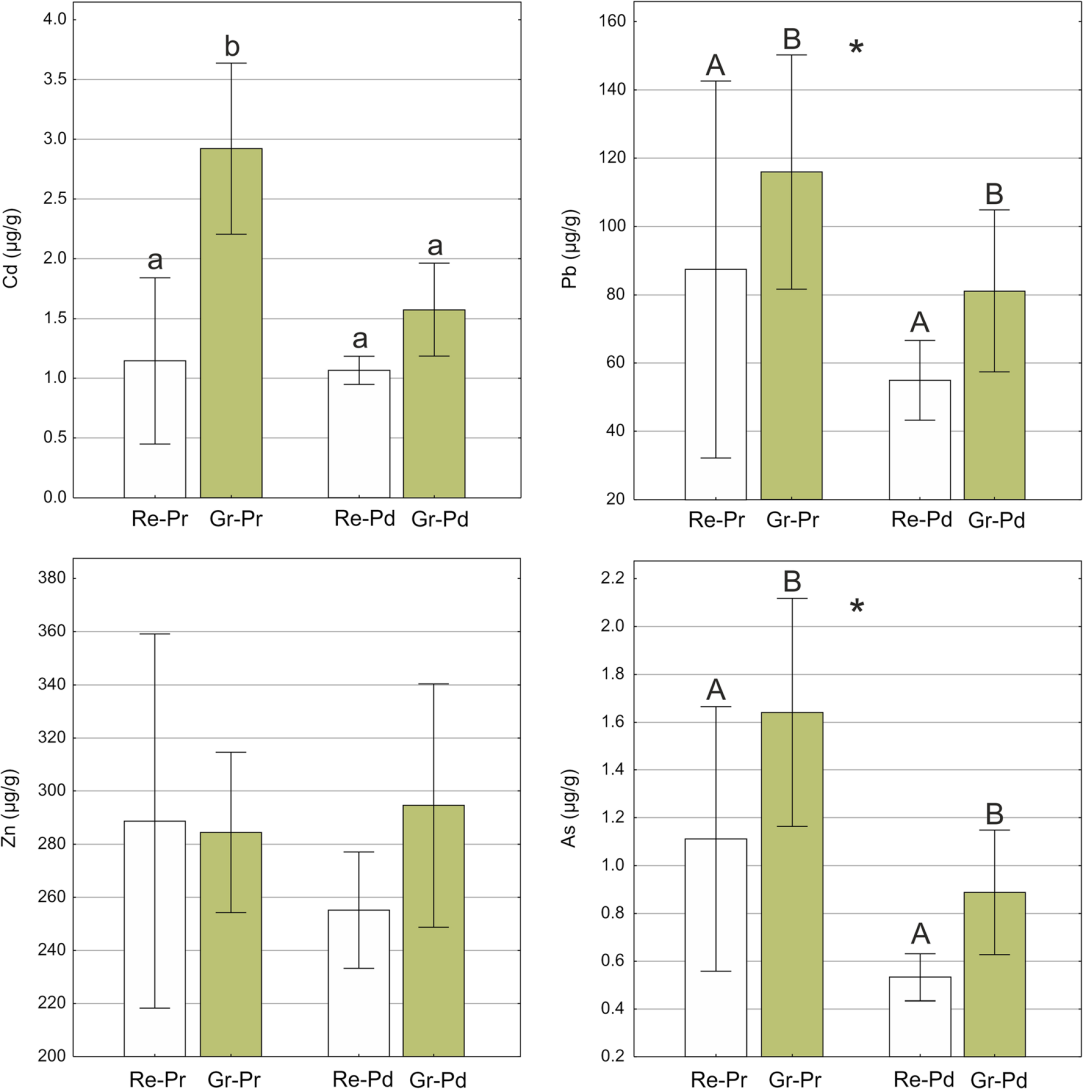
Concentrations of elements (Cd, Pb, Zn, As) in the lichen samples of *Cladonia cervicornis* subsp. *verticillata* classified according to the kind and form of thallus. Bars indicate mean values, and whiskers show minimum and maximum values. Filling refers to modified forms of thalli. Re-Pr, regular primary squamules; Gr-Pr, epigeic corticated granules; Re-Pd, typically formed regular podetia (secondary thallus); Gr-Pd, podetia covered with granules. Different letters above the bars indicate statistically significant differences (*p* < 0.05). The capital letters indicate a significant main effect of form of thallus and asterisks (*) indicate a significant main effect of kind of thallus. The lower-case letters indicate statistically significant interactions between kind and form of thallus. See Table S1 for details on the main effects and interactions

The concentration levels of the elements in lichen samples classified by kind and form of thallus are shown in graphs (Fig. 3). No significant impact on the Zn concentration (two-way ANOVA, *p* > 0.05) could be attributed to either kind or form of thallus. Nevertheless, the regular podetia showed a tendency towards lower Zn accumulation. The Cd content was significantly lower both in typical primary and secondary thalli, regardless of its form, than in granulose primary thalli (significant kind of thallus × form of thallus interaction, Table S1). The accumulation of Pb and As in primary thalli was significantly higher than in secondary thalli (significant kind of thallus effect). Moreover, granulose forms for both kinds of thalli accumulated Pb and As more effectively than typically developed, regular forms (significant form of thallus effect, Table S1).

### CL/CS factor

**Fig. 4:**
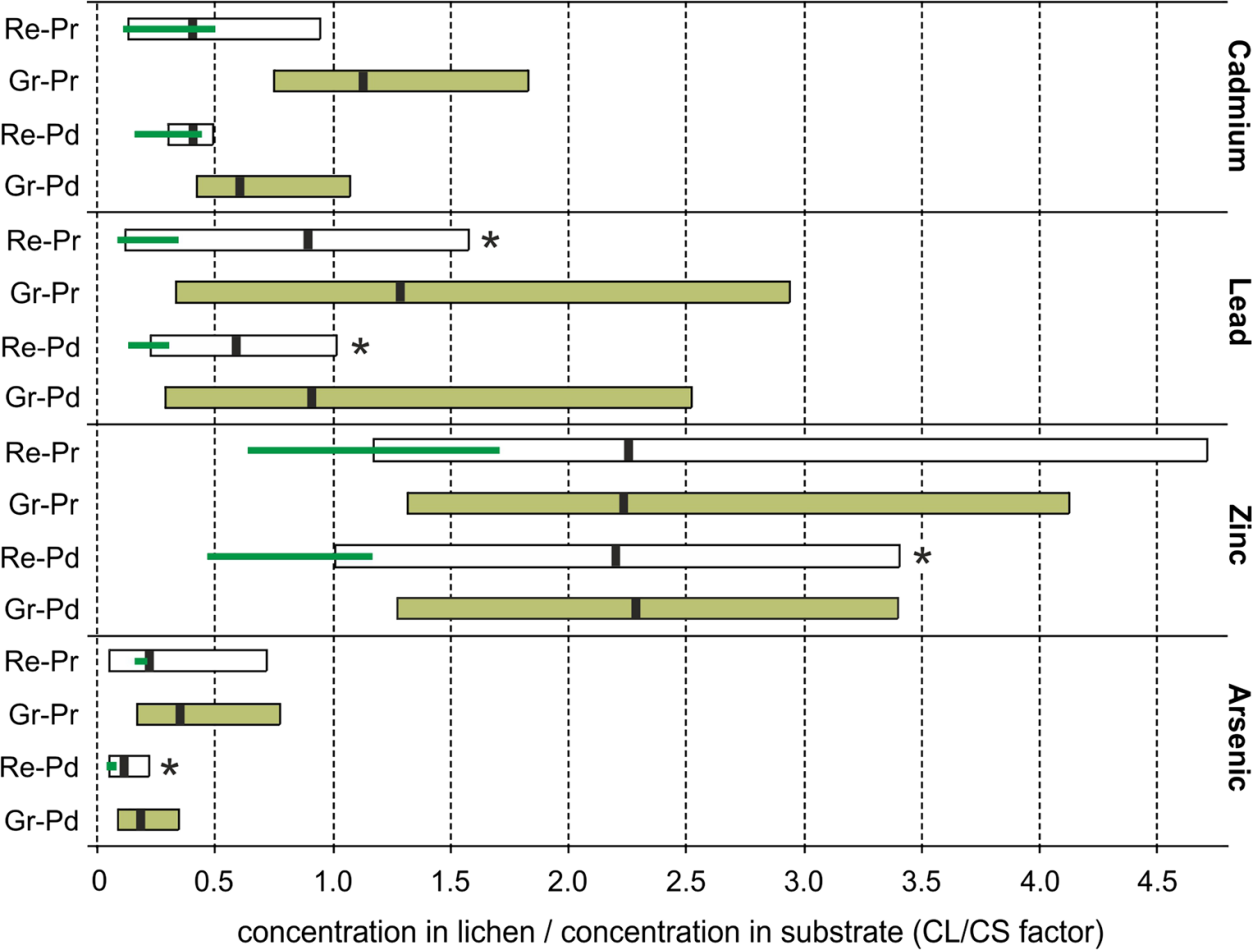
The ranges of the CL/CS factor for particular kinds and forms of the thallus of *Cladonia cervicornis* subsp. *verticillata* calculated as the ratio of element content in the thallus and the corresponding substrate. Filling refers to granulose forms of thallus; mean values are indicated by black vertical lines within the bars; green thin lines show the ranges for the samples from the reference area; asterisks (*) indicate significant differences (*p* < 0.05) between ratio values of samples from the Pustynia Starczynowska desert and the reference area according to the Mann-Whitney U-test. For abbreviations for forms of thallus, see the caption of Fig. 3 and Table 1

Mean and maximum values of the CL/CS factor for Cd, Pb and As were always higher in granulose forms of thallus irrespective of kind of thallus (Fig. 4). Nevertheless, a significant effect of both kind and form of thallus on the CL/CS factor was revealed only for Cd and As (Table S1). The CL/CS factor for Cd content was significantly lower both in squamulose primary thalli and secondary thalli regardless of form than in granulose primary thalli (significant kind of thallus × form of thallus interaction, Table S1), whereas the CL/CS factor for As for primary thalli was significantly higher than in secondary thalli (significant kind of thallus effect). Moreover, granulose forms of both kinds of thallus were characterised by a higher factor than typically formed primary and secondary thalli (significant form of thallus effect). Neither kind nor form of thallus had a significant impact on the CL/CS factor for Pb and Zn (two-way ANOVA, *p* < 0.05). Generally, analysed thalli of *Cladonia* demonstrated relatively weak element absorption from the substrate and low bioaccumulation tendencies in terms of Cd, Pb and As, since the mean values of the CL/CS factor usually did not exceed 1. The exceptions were granulose primary thallus samples in which the Pb and Cd concentration levels were slightly higher than in their host soil substrates. The accumulative response to the presence of Zn in the environment is quite different, as the mean values of the CL/CS factor exceeded 2 for all types of thallus samples from the Pustynia Starczynowska desert. Moreover, the minimum value of the calculated quotient for Zn was slightly above 1, and maximum values in the case of both forms of primary thallus exceeded 4. The ranges of the CL/CS factor obtained for the lichen samples from the reference area were always considerably lower than those for the samples from the Pustynia Starczynowska desert. Nevertheless, the comparative samples also achieved the highest CL/CS factor in relation to Zn accumulation (Fig. 4).

## Discussion

### Modifications of thallus structure

Structure of thallus in the form of corticate granules in no way corresponds to the taxa included in the so-called *C. cervicornis* group (see van Herk and Aptroot 2003; James 2009). Granulose primary thallus of *C. cervicornis* subsp. *verticillata* (Figs. 1a and 2a) is inconsistent with the regular primary squamules representative for this species. Instead of shapely podetia ending with shallow and centrally proliferating cups, abnormal excrescences of analogical granules occur in the upper part of the thallus (Fig. 1b). This *Cladonia* lichen in principle does not produce specialised vegetative propagules. However, the granules on podetia, irrespective of their origin, may fulfil this function, since they are not firmly attached to the surface and can be easily detached from the parental thallus (Fig. 1e). More subtle traits and differences in morphology were the basis for the recognition and description of many distinct *Cladonia* species. Subsequent molecular approaches confirmed that many of these indeed deserve the status of a separate species. On other hand, in numerous cases, it was found that the morphological diversities were in fact caused by the modelling influences of various environmental factors (e.g. Pino-Bodas et al. 2011). Molecular analysis revealed the DNA of only one fungus in all examined lichen samples. This proves that intraspecific morphological modifications of lichens can be substantial and may not concern only changes in particular traits but in fact may imply a change in the entire structure of the thallus.

The selection of a photosynthetic partner or partners by a fungus or by a group of photobionts in a lichen thallus and the sensitivity of prokaryotic and eukaryotic photobionts to pollutants have been the subjects of many studies (see e.g. Bačkor et al. 2010). It has been found that various photobionts are differentially sensitive to the presence of potentially toxic elements in the environment (Brown and Beckett 1983; Guschina and Harwood 2006; Paul and Hauck 2006). It is also assumed that the specificity of certain lichens for particular photobionts is linked to factors associated with the habitats in which these lichens exist (Werth and Sork 2010). Based on ITS algal sequences, we found no distinct differences in the diversity of photobionts among various types of thalli. All sequences relate to the genus *Asterochloris* which is typical for *Cladonia* lichens, including the species associated with affected and contaminated sites (Bačkor et al. 2010). Since our examination revealed no genetic background for morphological dissimilarities of thalli, we concluded that the luxuriant development of granules is most likely stimulated by habitat factors.

### Heavy crystalline deposit on thalli

Lichenised fungi can secrete oxalic acids as a metabolic by-product (Jones et al. 1980; Gadd et al. 2014). The SEM image shows fungal hyphae inside granules within a medulla heavily encrusted with small crystals (Fig. 2c) which are either isolated or, more frequently occur in groups (Fig. 2d, e). A detailed description of such crystals can be found in Jackson (1981). The functions of calcium oxalate in lichens remain unclear, and many possible roles have been considered, for instance, protective functions, structural strengthening, trapping and storage of calcium, and water and light regulation (Wadsten and Moberg 1985; Modenesi et al. 1998, 2000; Clark et al. 2001; Giordani et al. 2003). Salt production is a frequent feature of calcicolous lichens (Syers et al. 1967); accordingly, the synthesis of calcium oxalate has most often been interpreted as a detoxification process associated with excess Ca (Wadsten and Moberg 1985). In case of examined granulose forms of thalli, there is no real reason for calcium enrichment of the habitat as a stimulus for calcium oxalate production, since the content of this element in their host soil is basically negligible (Table 2). Much greater content of Ca was recorded at the reference area; however, the modified thalli were not observed. Control of water vapour, light gathering and reflection are additional important putative attributes of calcium oxalate that may be useful for lichens (Clark et al. 2001; Modenesi et al. 1998). They make sense in relation to the dry and sunlit habitat of the Pustynia Starczynowska desert. However, thick crystalline deposits occurred only on the thallial surfaces of granulose individuals growing in the vicinity of the smelter. Moreover, no accumulation of crystals occurs on the thalli covering the area with similar climate factors and genesis, but situated at a greater distance from the emitter. Although this oxalate is bound with Ca, its high accumulation has been observed in lichens under pollutant stress, especially in cases of SO_2_-polluted air. For example, thick and copious crystalline deposition relating to calcium oxalate was found on thallus surfaces in SO_2_-stressed and paraquat-treated specimens of *Parmotrema reticulatum*, a SO_2_-sensitive lichen species (Modenesi 1993). Notwithstanding, the phenomenon is not fully understood. Deposition of both whewellite and weddellite, in clean-air sites as well as in moderately and heavily polluted areas, was reported by Garty et al. (2002); this suggests that the presence of air pollutants is not necessarily the basic determinant for crystal formation. Some researchers have suggested that calcium oxalate crystals could contribute to heavy metal detoxification (Nakata 2003; Mazen 2004); other studies showed that they have no direct effect on this process (e.g. Dou et al. 2009). Besides calcium binding, oxalic acid secreted by the lichen mycobiont acts as a chelator of metal ions (Seaward et al. 1989; Sarret et al. 1998; Gadd et al. 2014). We cannot exclude the supporting role of calcium oxalate in the disposal of heavy metals or the presence of metal oxalates in the granulose specimens, since element detection by EDX, in addition to Ca, counted Pb in places saturated with calcium oxalate crystals, especially tetragonal prisms (Fig. S2). Paul et al. (2003) found that Mn was incorporated in calcium oxalate crystals on the surfaces of the hyphae of *Hypogymnia physodes*. We assume that harmful effects resulting from smelter activity in the form of metal dust fallout and SO_2_ emission at least enhances crystalline deposition in the examined lichen thalli. On the other hand, it is also probable that the parts of thalli, with fungal hyphae showing increased oxalate production activity, are secondary transformed into granulose form as the result of high internal condensation of crystalline deposits. This is supported by the fact that the granulose deformations anatomically constitute a coherent structure within podetia (Fig. 1d, f), while heavy crystalline deposits occur only in the deformed parts of thalli.

### Heavy-metal accumulation in lichen thalli

Considering the matter broadly, the examined specimens accumulated heavy metals at a lower level than samples of *Cladonia* studied from the same industrial area (cf. Pawlik-Skowrońska et al. 2008; Osyczka and Rola 2013c); although, direct comparison is not possible due to considerable differences in the contamination of the lichens’ host substrate. Due to the morphological duality of the genus, element accumulation can be regarded in three ways: content in primary squamules, in the secondary thallus and jointly, in both kinds of thalli. Previous studies showed that differences in particular element contents may be considerable (see Osyczka et al. 2016). We recorded significant effects of both kind and form of thallus (Table S1) on the accumulation of xenobiotic elements, i.e. Cd, Pb and As. Generally, the burdens of these elements in the primary kind and granulose form of thallus are considerably higher than in the secondary and regular forms. The squamules are in intimate contact with the host substrate and are frequently devoid of the cortex layer on the bottom. This results in potential direct element uptake from the substrate and strong substrate contamination constitutes the main source of elements in the thallus (Lawrey and Rudolph 1975; Osyczka et al. 2016). The content of Zn in the thalli showed no apparent regularity and no significant effect was recorded. Nevertheless, regular podetia were characterised by the lowest mean Zn concentration of all thallus types. The higher concentrations of elements in granulose forms are probably associated with a greater exchange surface per area and mass unit in comparison with regular forms. This may explain the greater uptake of elements directly from the atmosphere and intensified trapping of particulate matter in the intercellular spaces of the thallus (Tretiach et al. 2005; Bertuzzi and Tretiach 2013). Even slight differences in morphological appearance within the same species could result in differing accumulation capacities (see also Goyal and Seaward 1982; Otnyukova 2007).

When considering bioaccumulation in epigeic lichens, two vectors for delivery of elements are possible: air and the host substrate. If substrate contamination is very high and without doubt exceeds atmospheric deposition, accumulation in epigeic lichens must largely depend on element enrichment from the corresponding substrate (Bargagli et al. 1987; Chettri et al. 1997; Bačkor and Fahselt 2004). The matter becomes more complicated when the most important source of particular elements is not easy to define. *Cladonia* lichens have been recognised as weak accumulators of heavy metals in relation to soil contamination (Pawlik-Skowrońska et al. 2008; Osyczka and Rola 2013c), and reported BAF values were, as a rule, considerably lower than 1 (Osyczka et al. 2016). This also applies roughly to most of the examined samples. The evident exception is Zn for samples from the Pustynia Starczynowska desert and Cd and Pb for some samples of the granulose form of thalli (Fig. 4). Additionally, it turned out that all ranges of the CL/CS ratios for the samples of both kinds of thallus from the reference area are much lower than those obtained from the Pustynia Starczynowska desert (Fig. 4). Comparison of the CL/CS ratios from two areas (Pustynia Starczynowska vs Pustynia Błędowska) with relatively similar soil contamination but lower air pollution in the reference area gives a strong ground for believing that the main load of elements in the thalli collected in the vicinity of the zinc smelter was associated with local atmospheric fallout. Therefore, the accumulation of heavy metals in the epigeic *Cladonia* lichens may be indicative of air pollution. This could be applicable to the areas adjacent to emission source in which epiphytic lichens, commonly used as bioindicators, do not exist.

## Conclusions

The phenotypic plasticity of some *Cladonia* species may take on an intensified character and consequently may lead to the emergence of features that essentially are not attributes of that species in the context of its morphological definition. Heavy crystalline deposits are limited to granulose parts of the examined *Cladonia* thalli. However, it remains questionable whether the intensive production of crystals initiates the thalli deformations or whether the granules constitute a specialised structure in which crystals are accumulated.

Taking into consideration previous reports on the low bioaccumulation capacity of *Cladonia* in relation to the host substrate, a ratio of element concentrations in the lichen thallus to those in the substrate (CL/CS factor) greater than 1 clearly suggest that a substantial part of the element load in the thallus originates from atmospheric fallout. Consequently, the accumulation of heavy metals in the epigeic *Cladonia* lichens may be alternatively indicative of air pollution.

Morphological modifications within the same lichen species could greatly affect heavy-metal accumulation in variously formed individuals. This has implications for bioindication and biomonitoring studies in which the concentration of a given element in the thalli is considered as a determinant of the environment condition. Selecting morphologically uniform samples and trying to avoid collecting variously formed thalli are simple ways to eliminate at least one of the factors that may yield undesirable and hidden artefacts. Thus, this procedure is desirable for inclusion in biomonitoring sampling protocols.

## Electronic supplementary material

The online version of this article contains supplementary material, which is available to authorized users.

ESM 1 (PDF 939 kb)
