# Supplementary material for: Modifications in the structure of the lichen Cladonia thallus in the aftermath of habitat contamination and implications for its heavy-metal accumulation capacity
Source: Environ Sci Pollut Res Int. 2017 Nov 5;25(2):1950–61. doi: 10.1007/s11356-017-0639-1 (PMC5766729; doi:10.1007/s11356-017-0639-1)
Supplement: Supplementary file 1 — (PDF 939 kb) [file 11356_2017_639_MOESM1_ESM.pdf]

SUPPLEMENTARY DATA TO THE ARTICLE:

Modifications in the structure of the lichen *Cladonia* thallus in the aftermath of habitat contamination and implications for its heavy-metal accumulation capacity

Piotr Osyczka[a], Piotr Boroń[b], Anna Lenart-Boroń[c], Kaja Rola[a,*]

[a]*Institute of Botany, Faculty of Biology and Earth Sciences, Jagiellonian University, Kopernika 27, 31-501 Kraków, Poland*
[b]*Department of Forest Pathology, Mycology and Tree Physiology, University of Agriculture in Kraków, 29 Listopada Ave. 46, 31-425 Kraków, Poland*
[c]*Department of Microbiology, University of Agriculture in Kraków, Mickiewicza Ave. 24/28, 30-059 Kraków, Poland*

* *Corresponding author*. Tel.: +48 12 6633652; fax: +48 12 4230949
E-mail address: kajaskubala@interia.pl (Kaja Rola)

Fig. S1 The results of 50% majority-rule Bayesian analysis of newly-sequenced and selected GenBank-sourced ITS sequences of *Cladonia*. The values of posterior probabilities are indicated on the branches.

Re-Pr – regular primary squamules;
Gr-Pr – epigeic corticated granules;
Re-Pd – typically formed regular podetia;
Gr-Pd – podetia covered with granules;
(A) – lichen samples with detected *Asterochloris* photobiont.

.

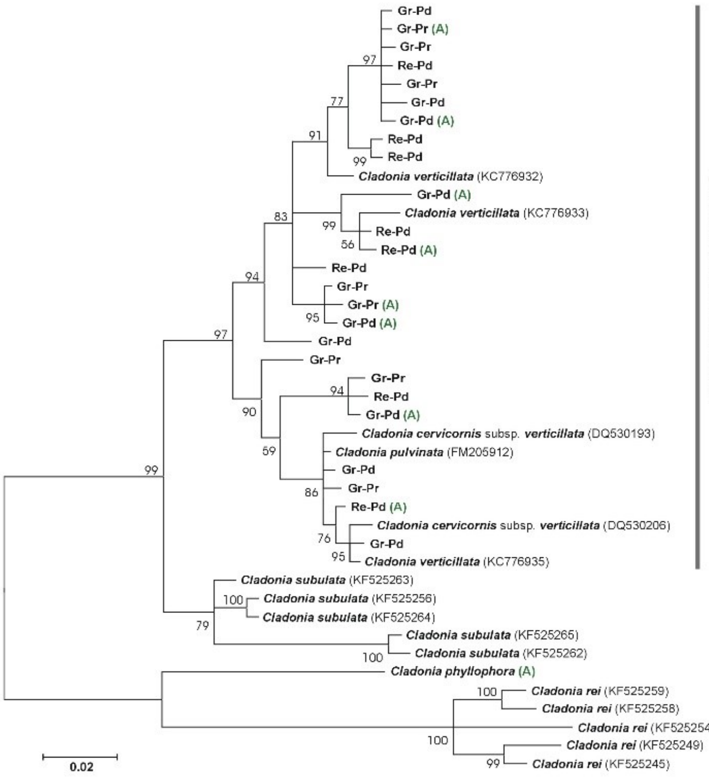

Fig. S2 Exemplary energy-dispersive X-ray spectroscopy (EDX) spectra obtained from areas with heavy oxalate deposition on the surface of corticate granules. **A** – aggregate of bipyramids; **B** – a crater-like structure filled with short tetragonal prisms.

A

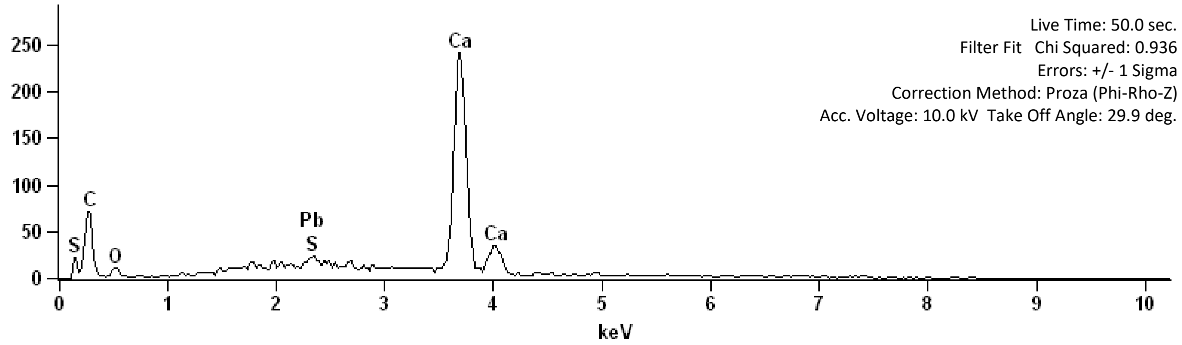

Quantitative Results

Element Line	Net Counts	NetCounts Error	Weight%	Weight% Error	Atom%	Atom% Error	Formula	Compnd%	#Cations
O K	64	+/- 64	7.99	+/- 1.00	18.26	+/- 2.28	O	7.99	---
S K	91	+/- 26	1.20	+/- 0.34	1.37	+/- 0.39	S	1.20	---
Ca K	3212	+/- 86	87.53	+/- 2.34	79.79	+/- 2.14	Ca	87.53	---
Pb M	120	+/- 47	3.28	+/- 1.28	0.58	+/- 0.23	Pb	3.28	---
Total			100.00		100.00			100.00	0.000

B

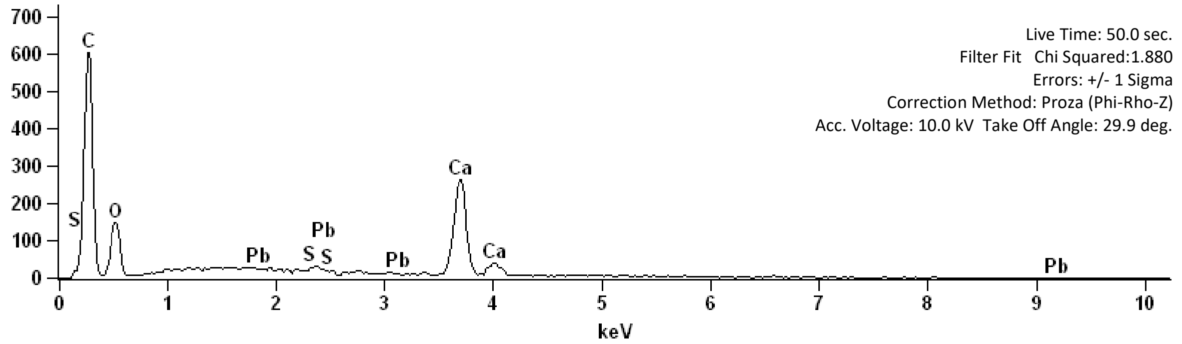

Quantitative Results

Element Line	Net Counts	NetCounts Error	Weight%	Weight% Error	Atom%	Atom% Error	Formula	Compnd%	#Cations
O K	1132	+/- 40	46.17	+/- 1.63	69.42	+/- 2.45	O	46.17	---
S K	4	+/- 30	0.03	+/- 0.21	0.02	+/- 0.16	S	0.03	---
Ca K	3462	+/- 92	50.22	+/- 1.33	30.14	+/- 0.80	Ca	50.22	---
Pb M	244	+/- 54	3.58	+/- 0.79	0.42	+/- 0.09	Pb	3.58	---
Total			100.00		100.00			100.00	0.000

Table S1 Results of two-way ANOVA ($p<0.05$) for the effects of kind of thallus (primary and secondary), form of thallus (granulose and regular) and their interaction on the element concentrations and CL/CS factors (i.e. concentration of the element in the lichen thallus ÷ concentration of the element in the corresponding substrate). The effects in bold are statistically significant.

Parameters	Element concentration						CL/CS factor					
	Kind of thallus		Form of thallus		Kind of thallus × Form of thallus		Kind of thallus		Form of thallus		Kind of thallus × Form of thallus	
	F	p	F	p	F	p	F	p	F	p	F	p
Cd	**17.50**	**<0.001**	**44.84**	**<0.001**	**13.74**	**0.001**	**13.37**	**<0.001**	**37.32**	**<0.001**	**11.43**	**<0.001**
Pb	**9.23**	**0.004**	**6.06**	**0.019**	0.01	0.919	3.26	0.079	3.66	0.064	0.01	0.913
Zn	0.65	0.426	1.461	0.235	2.25	0.142	0.12	0.727	0.18	0.677	0.29	0.597
As	**28.99**	**<0.001**	**12.81**	**0.001**	0.49	0.490	**9.32**	**0.004**	**4.13**	**0.049**	0.35	0.559
